# Molecular mechanisms underlying nuchal hump formation in dolphin cichlid, *Cyrtocara moorii*

**DOI:** 10.1038/s41598-019-56771-7

**Published:** 2019-12-30

**Authors:** Laurène Alicia Lecaudey, Christian Sturmbauer, Pooja Singh, Ehsan Pashay Ahi

**Affiliations:** 1 Institute of Biology, University of Graz, Universitätsplatz 2, A-8010 Graz, Austria; 2 Department of Natural History, NTNU University Museum, Norwegian University of Science and Technology, NO-7491 Trondheim, Norway; 3 Institute of Biological Sciences, University of Calgary, 2500 University Dr NW, Calgary, AB T2N 1N4 Canada; 4 Department of Comparative Physiology, Uppsala University, Norbyvägen 18A, SE-75 236 Uppsala, Sweden

**Keywords:** Biological techniques, Reverse transcription polymerase chain reaction, Evolution, Coevolution, Evolutionary developmental biology, Molecular evolution, Transcription, Transcriptomics

## Abstract

East African cichlid fishes represent a model to tackle adaptive changes and their connection to rapid speciation and ecological distinction. In comparison to bony craniofacial tissues, adaptive morphogenesis of soft tissues has been rarely addressed, particularly at the molecular level. The nuchal hump in cichlids fishes is one such soft-tissue and exaggerated trait that is hypothesized to play an innovative role in the adaptive radiation of cichlids fishes. It has also evolved in parallel across lakes in East Africa and Central America. Using gene expression profiling, we identified and validated a set of genes involved in nuchal hump formation in the Lake Malawi dolphin cichlid, *Cyrtocara moorii*. In particular, we found genes differentially expressed in the nuchal hump, which are involved in controlling cell proliferation (*btg3*, *fosl1a* and *pdgfrb*), cell growth (*dlk1*), craniofacial morphogenesis (*dlx5a*, *mycn* and *tcf12*), as well as regulators of growth-related signals (*dpt*, *pappa* and *socs2*). This is the first study to identify the set of genes associated with nuchal hump formation in cichlids. Given that the hump is a trait that evolved repeatedly in several African and American cichlid lineages, it would be interesting to see if the molecular pathways and genes triggering hump formation follow a common genetic track or if the trait evolved in parallel, with distinct mechanisms, in other cichlid adaptive radiations and even in other teleost fishes.

## Introduction

Given the striking adaptive morphological diversity of craniofacial structures in teleost fish, it comes with no surprise that these differences in naturally occurring systems have garnered considerable attention in studies of developmental and molecular biology, beyond models like zebrafish^1,2^. Myriad molecular players and interconnected signalling pathways, which participate in development and morphogenesis of craniofacial musculoskeletal structures, are described in teleost fish^3,4^. However, when it comes to craniofacial soft tissues, little is known about the morphogenic molecular factors and underlying signals. In cichlids for instance, researchers have only recently attempted to investigate the potential molecular mechanisms forming soft-tissue traits such as enlarged lips and nose flaps^5–9^. Such extreme and novel phenotypes are commonly referred to as exaggerated traits and are thought to have contributed to the evolutionary success of the cichlid adaptive radiation.

Another exaggerated craniofacial phenotype that is observed in different groups of teleost, including cichlid fishes, is the overgrowth and protrusion of soft and possibly underlying hard tissues on the forehead, called the nuchal hump, also known as forehead swelling^10–15^. In the Central American cichlid species, *Amphilophus citrinellus*, which is well-known for its large nuchal hump (in both sexes), the hump tissue seems to develop in response to hormonal stimulus and contain modified nuchal hypodermis and high amount of fat stored in the same tissue^10^. Similar nuchal humps have evolved in parallel in African cichlids, which shared a common ancestor with the South American cichlids ~100 MYA^16,17^. Both species of the Lake Tanganyika endemic tribe Cyphotilapiini (*Cyphotilapia gibberosa*, *Cyphotilapia frontosa* and *Ctenochromis benthicola*) have nuchal humps, as well as the species *Cyrtocara moorii* from the hyperdiverse radiation of the tribe haplochromini in Lake Malawi^17^. The nuchal hump in *Cyphotilapia gibberosa* is also formed by hypertrophy of hypodermis and excessive fat storage of in this tissue^18^. The exact function of the nuchal hump in fish is still a subject of debate; however, a few plausible hypotheses have been proposed. For instance, its potential role in sex recognition (in dimorphic species), species recognition, mechanical advantage in a fight, improved hydrodynamics and anti-predation role^11,13,18^.

The most speciose adaptive radiation of cichlids is found in Lake Malawi with ~800 species^19^ that diverged <5 MYA^20^. However, new estimates place this age closer to ~ 0.7–0.8 MYA^21^, which corresponds to its last continuous extended deep-lake phase. The ecological and morphological diversity in this lake was driven by repeated lake-level fluctuations between 1.2–0.8 MYA, that opened up novel niches facilitating bouts of divergence and secondary contact (Ivory *et al*. 2016). Thus, ecological specializations are largely comprised of adaptations to benthic (sand and rocky shores) and pelagic (open water) habitats, as well as deep-waters. Specifically, these adaptations occurred in three stages to (a) different habitats (b) different food sources within each habitat and (c) diversification in body colour associated with sexual selection^20^. Cichlids from Lake Malawi are therefore an excellent model to study unique and parallel exaggerated craniofacial traits such as nose flap^22^, hypertrophied lips^23^, and nuchal hump^17^.

**Figure 1:**
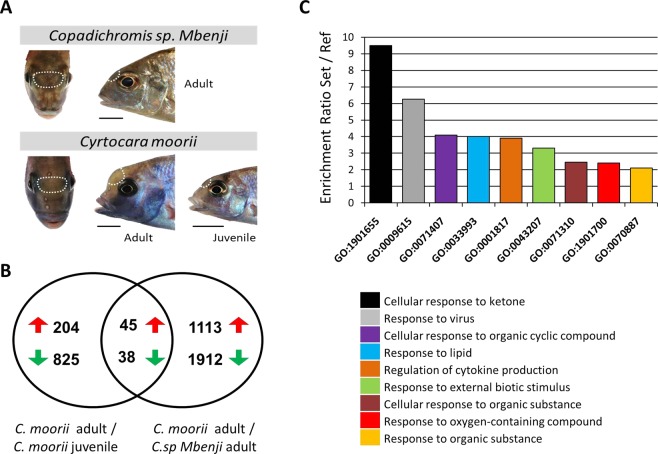
Nuchal hump tissue samples and general information about differentially expressed genes identified through RNA-seq. (**A**) The white dashed lines circumscribe the nuchal regions specifically dissected for RNA sampling. (**B**) A Venn diagram showing 87 genes common between the differentially expressed lists of genes in the nuchal regions (between the two comparisons), and among them, 45 had increased expression while 38 decreased expression in the nuchal hump of adult *C. moorii*. (**C**) Gene ontology enrichment analysis for biological processes, using the shared 87 differentially expressed genes, based on Manteia tool (minimum 4 genes in each GO term).

The aim of this study was to identify genes with potential role in the formation of nuchal hump soft tissues in *Cyrtocara moorii*, to better understand its evolution. This species is a member of Haplochromine cichlid tribe, endemic to Lake Malawi in East Africa, which develops conspicuous hump-headed morphology in adult males^17,24,25^ (Fig. 1A). To reach this goal, we set out to profile the gene expression changes in the nuchal hump soft tissues of young adult males of *C. moorii* using RNA-seq method. As a control, we used soft tissues from the nuchal region of juvenile males of *C. moorii*, prior to the emergence of the hump. In addition, to exclude genes which are differentially expressed during later phases in the adult ontogeny and also to have a profile of interspecies comparison, we used soft tissues from the nuchal region of adult males of *Copadichromis sp Mbenji*, a closely related Haplochromine species without hump-headed morphology^26^ (Fig. 1A). Both *C. moorii* and *C. sp Mbenji* belong to two closely related genera within non-Mbuna group of Haplochromine cichlids in Lake Malawi^27,28^. Finally, we carried out qPCR expression analysis of a selected set of most promising candidate genes identified by RNA-seq, in order to validate our findings from the transcriptome dataset. To our knowledge, this study provides the first transcriptional profile of the nuchal hump in fish, and the list of identified differentially expressed genes in the nuchal hump tissues allowed us to hypothesize potential molecular mechanisms controlling the formation of this craniofacial structure reported in different groups of teleost fish.

## Results

### RNA-seq and differential gene expression in nuchal hump

The transcriptome sequencing yielded between 6.04 for C.sp-A2 to 11.8 C.m-J4 million reads per sample (Table 1). Throughout the paper we respectively refer to *C. moorii* at young adult and late juvenile stages as C.m-A and C.m-J, and to *C. sp Mbenji* at young adult stage as C.sp. The raw sequence reads have been submitted to the Sequencing Read Archive (SRA) of NCBI (accession number: PRJNA545415). After quality filtering, each sample had between 6.02 and 11.7 million reads (Table 1). It is worth mentioning that this range of quality filtered reads per sample is enough to estimate differential expression of moderate to high expressed genes, however, this might not be optimal to identify differential expression of low expressed genes. The comparison between the nuchal regions of adult and juvenile *C. moorii* (C.m-A vs C.m-J) resulted in 1112 differentially expressed (DE) genes out which 249 genes showed increased expression in the nuchal hump (C.m-A) and 863 genes had reduced expression this region (Fig. 1B)(Supplementary Data 1). The number of DE genes between the nuchal regions of adult *C. moorii* and *C. sp. Mbenji* (C.m-A vs C.sp) was 3108 and among them 1158 genes had increased expression in the nuchal hump (C.m-A) whereas rest of the genes showed reduced expression in this region (Fig. 1B). The total number of overlapping DE genes between the two comparisons was 83, and except for one gene, *muc5ac*, all the genes had either higher or lower expression in C.m-A (nuchal hump) than the two other groups (C.m-J vs C.sp) (Fig. 1B)(Supplementary Data 1). This clearly indicates that the two sampling groups without frontal hump, even though they are from different species and developmental stages, have more similar expression pattern than the sample group with the nuchal hump, in regard to the overlapping DE genes. Thus, it is highly probable that most of the overlapping DE genes are involved in the formation of nuchal hump phenotype in adult males of *C. moorii*.

**Table 1:** Number of RNA sequencing reads obtained for each sample.

Sample	Raw PE reads	Quality trimmed PE reads
Cm-A1	9,494,727	9,424,118
Cm-A2	6,246,029	6,222,606
Cm-A3	9,677,079	9,628,558
Cm-A4	7,266,067	7,242,052
Cm-A5	7,907,752	7,882,215
Cm-J1	10,178,413	10,151,144
Cm-J2	9,962,062	9,933,590
Cm-J 3	9,847,497	9,818,416
Cm-J 4	11,820,465	11,779,300
Cm-J 5	11,503,337	11,476,285
CSp-A1	7,070,511	7,050,501
CSp-A2	6,040,697	6,015,816
CSp-A3	7,465,114	7,439,942
CSp-A4	9,916,715	9,888,063
CSp-A5	7,445,224	7,405,088

Next, we performed gene ontology enrichment analysis using the same set of overlapping DE genes. Within the significantly enriched GO terms with highest enrichment ratios, we found cellular and molecular responses to a variety of organic compounds to be overrepresented (Fig. 1C). These processes include responses to ketone, lipid, cyclic and oxygen-containing compounds, and on the other hand, molecular processes involved in cellular response to external stimuli such biotic elements and viruses appeared to be significantly enriched. Importantly, a molecular process involved in regulation of cytokine production was also found among the enriched GO terms (Fig. 1C). These findings suggest differential activation of multiple molecular processes, which are mainly associated with responses to different internal and external molecular compounds or biotic elements in the developing nuchal hump of adult *C. moorii*.

**Figure 2:**
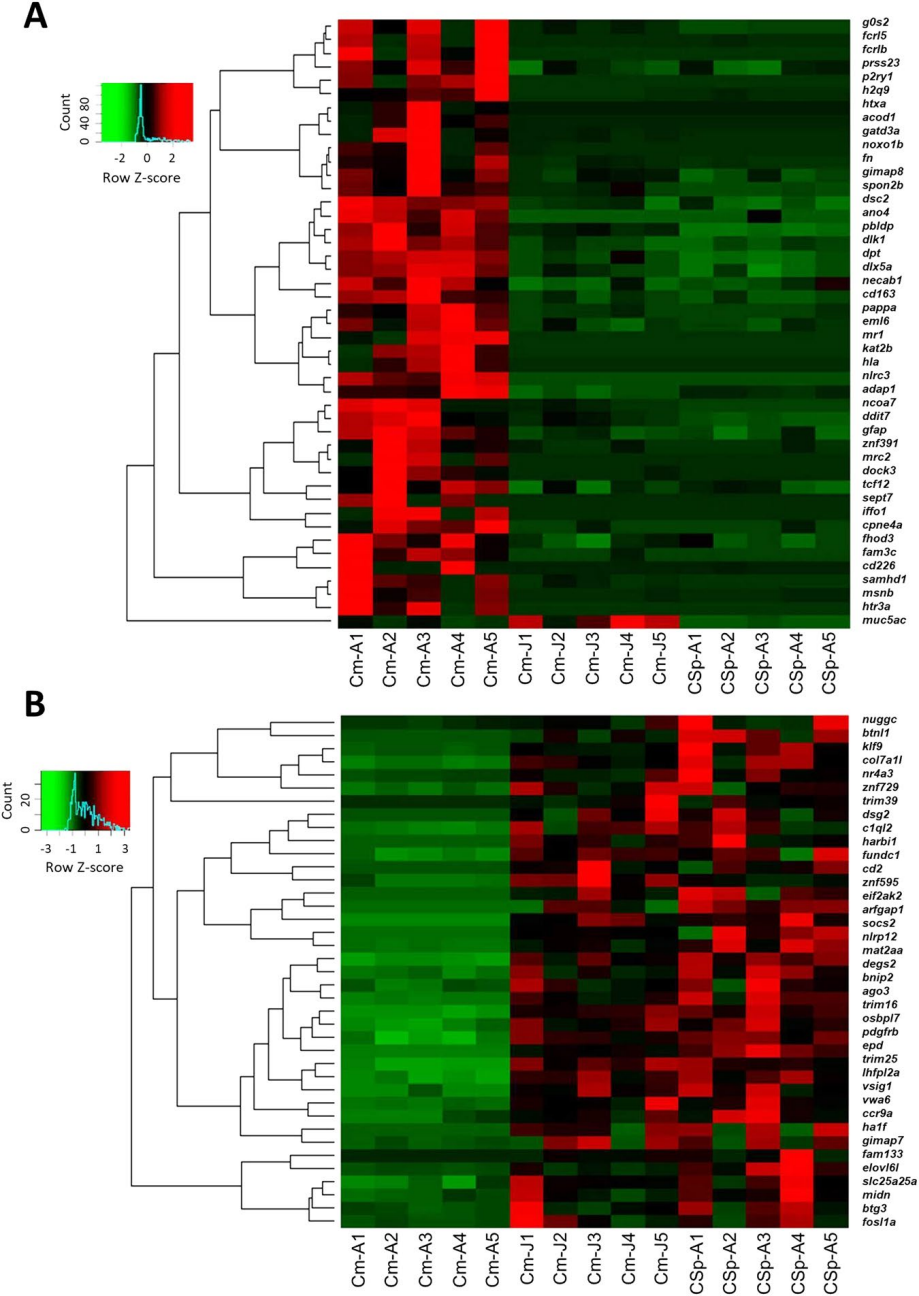
Differentially expressed genes in the nuchal hump region of *Cyrtocara moorii* identified through RNA-Seq. Dendrogram clusters of genes with increased (**A**) and decreased (**B**) expression in the nuchal hump region of adult *C. moorii* when compared to the corresponding tissues in juvenile *C. moorii* and adult *C. sp Mbenji*. Red and green shadings indicate higher and lower relative expression, respectively.

Out of 83 overlapping DE genes, 45 had increased expression and 38 genes had reduced expression in the nuchal hump (Fig. 1B). Two separate dendrogram-heatmaps clustering of the DE genes (one with the set of genes higher expressed in the nuchal hump and one for the set of genes lower expressed) revealed at least 4 major clusters of genes with similar expression pattern in each of the gene group (Fig. 2). The existence of multiple clusters demonstrates that the expression patterns of the DE genes vary even within the groups of genes with increased or decreased expression in the nuchal hump. This might indicate potentially distinct upstream factors regulating the expression of each gene cluster. However, it should be noted that the differential expression for some of the gene clusters were not consistent across all individual samples. Among the gene clusters, we found several transcriptional regulators with consistent expression differences across the samples such as *ago3*, *dlk1*, *dlx5a*, *mycn*, *socs2* and *tcf12*. The function(s) of most of the DE genes are not known in relation to craniofacial morphogenesis, but at least 12 genes have already been shown to have related functions in vertebrates (Table 2). Therefore, we selected them for further expression analysis with qPCR, in order to confirm their differential expression in the nuchal hump.

**Table 2:** Selected differentially expressed genes, identified by RNA-seq in the nuchal hump tissue with potential related functions in vertebrates.

Gene	Related function	Organism	References
*ago3*	A post-transcriptional regulator involved in facial morphogenesis including forehead, nasal and palpebral tissues	Human	^57^
*btg3*	An anti-proliferative protein with potential role in size determination of facial elements including forehead, ear and nose	Human	^93^
*dlk1*	A regulator of cell growth involved in craniofacial morphogenesis including skull, jaw, face, nose, forehead, lips and ears	Human Mice	^79,80^
*dpt*	A non-collagenous matrix protein and enhancer of TGF-beta activity involved in keratinocyte migration, angiogenesis and cell adhesion	Human Mice	^83–85^
*cd163*	A histiocytic lineage marker involved in benign neoplasm of facial soft tissues and xanthogranuloma	Human	^81,82^
*dlx5a*	A homeobox transcription factor involved in frontal bone development and craniofacial morphogenesis with various effects on facial soft tissues	Human Mice Chicken Zebrafish	^68–71^
*fosl1a*	A FOS protein regulating cell proliferation and differentiation involved in skeletal effects of Pfeiffer syndrome with craniofacial deformities including fronto-orbital advancement	Human	^94,95^
*mycn*	A regulator of apoptosis and autophagy involved craniofacial morphogenesis with effects on skull and frontal bone development	Human Mice	^59–61^
*pappa*	An enzyme cleaving IGF-binding proteins with effects on craniofacial skeletogenesis including frontal bone and skull development	Mice	^89^
*pdgfrb*	A receptor of PDGF involved in craniofacial development with effects on nose, jaw and forehead morphogenesis	Human Mice Zebrafish	^37–41^
*socs2*	A suppressor of cytokine signaling which limits IGF and GH signals and negatively regulates skeletal and dermal growth	Human Mice	^62–66^
*tcf12*	A transcription factor involved in cranial skeletal development involved in frontal bone morphogenesis and cranial vault thickening	Human Mice	^74–76^

### Expression validation using qPCR

The accuracy of gene expression quantification by qPCR strongly relies on stably expressed reference genes^29^, and based on our previous observations in East African cichlids, every experimental condition, developmental stage, tissue, and species require validation of different reference gene(s)^30–35^. To select candidate reference genes, we ranked the genes with no expression difference (FDR = 1) in each of the RNA-seq comparisons, based on their coefficient variation (CV) across all the samples. The top 7 genes with lowest CV were chosen as candidate reference genes for qPCR expression analysis. None of the candidate have been identified as suitable reference genes in previous studies of East African cichlids, indicating the importance of identification of suitable reference genes for each experimental setup^30–34,36–38^. The results of reference gene rankings by geNorm and NormFinder software suggested *rnf123* and *cnnm3* as top two most stable reference genes (Table 3). The BestKeeper results, however, differed slightly from the two other software, in which *cnnm3* and *rn123* were ranked second and third, respectively, based on *r* values. In another BestKeeper ranking (by SD values), *rnf123* and *cnnm3* appeared to be second and fifth, respectively. Taken together, *rnf123* and *cnnm3* seemed to be the only genes that were ranked consistently among the most stable reference genes, and therefore, we used the average expression of *rnf123* and *cnnm3* in order to normalize the expression values of selected target genes in the next step.

**Table 3:** Ranking of candidate reference genes across the nuchal tissues using three different software.

BestKeeper				geNorm		NormFinder	
Ranking	SD	Ranking	r	Ranking	M	Ranking	SV
*trappc12*	0.493	*ncor2*	0.984	*cnnm3*	0.244	*rnf123*	0.087
*rnf123*	0.495	*cnnm3*	0.98	*rnf123*	0.261	*cnnm3*	0.091
*hnrnpa1*	0.524	*rnf123*	0.968	*zhx3*	0.263	*zhx3*	0.107
*cnnm3*	0.549	*smarcc1*	0.967	*trappc12*	0.269	*ncor2*	0.108
*zhx3*	0.612	*zhx3*	0.965	*ncor2*	0.273	*trappc12*	0.111
*ncor2*	0.659	*trappc12*	0.963	*hnrnpa1*	0.292	*hnrnpa1*	0.116
*smarcc1*	0.683	*hnrnpa1*	0.954	*smarcc1*	0.301	*smarcc1*	0.120

Abbreviations: SD = Standard deviation, r = Pearson product-moment correlation coefficient, SV = Stability value, M = Mean value of stability.

**Figure 3:**
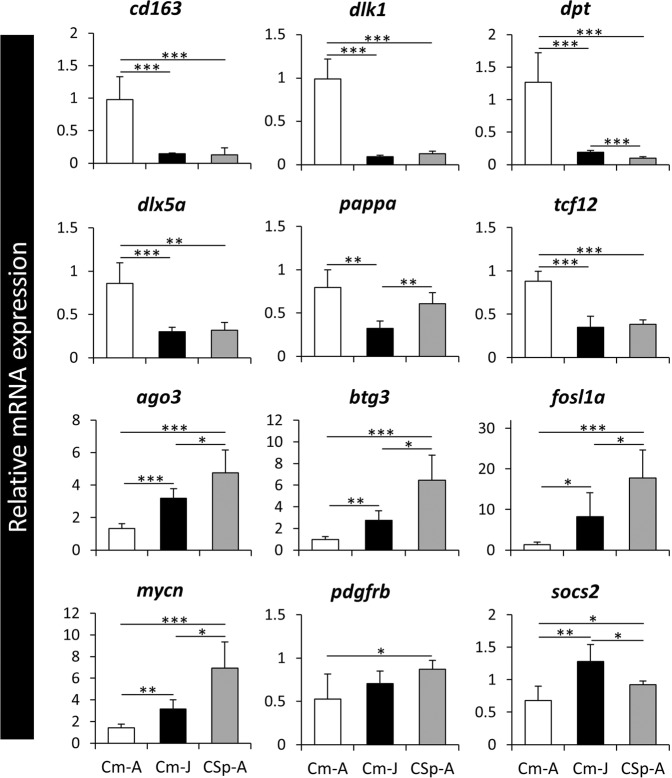
Expression analysis of a selected set of identified genes through qPCR. The bars represent means and standard deviations of RQ values for five biological replicates in each nuchal region. The statistical differences are shown by one, two and three asterisks above bars indicating *P* < 0.05, 0.01 and 0.001, respectively.

Among the identified DE genes by RNA-Seq, we chose 12 genes with known role in craniofacial morphogenesis of soft and/or hard tissues in vertebrates (Table 2), to be examined by qPCR to validate our transcriptome data analysis (Fig. 3). In the RNA-seq results, 6 of the selected candidate genes have shown increased expression whereas the other 6 genes had reduced expression level in the nuchal hump of adult *C. moori*. Almost all of the genes with increased expression in the nuchal hump by RNA-seq, *cd163*, *dlk1*, *dpt*, *dlx5a*, and *tcf12* also showed higher expression in Cm-A than Cm-J and CSp-A by qPCR, except for *pappa*, but only the comparison between Cm-A and CSp-A (Fig. 3). Similarly, all of the genes with reduced expression in the nuchal hump by RNA-seq, *ago3*, *btg3*, *fosl1a*, *mycn* and *socs2*, also displayed lower expression in Cm-A than Cm-J and CSp-A by qPCR, except for *pdgfrb*, but only the comparison between Cm-A and Cm-J (although such a tendency was observed in the qPCR data) (Fig. 3). The high degree of consistency between RNA-seq and qPCR results confirms the validity of our transcriptome data analysis in this study.

## Discussion

Delineating the molecular basis of ecologically important traits is a crucial step towards understanding their evolution and function. In this study, we focused on the nuchal hump in cichlids that is an exaggerated soft-tissue craniofacial trait, hypothesised to impart some adaptive advantage. However, little is known about nuchal hump development in cichlids, even though this extreme phenotype has evolved repeatedly in several lakes, across different continents. Using gene expression profiling, we identified a set of genes that shed light on the molecular pathways involved in the nuchal hump formation in the dolphin cichlid, *C. moorii*. To do this, we used tissues in the nuchal area of juvenile *C. moorii* which had not yet developed the hump in this area, in order to identify a list of DE genes potentially involved in the formation of nuchal hump. To filter out the age-dependent DE genes, we used the nuchal tissues from adult males of another closely related species, *C. sp Mbenji*, which does not form any hump, in the comparable nuchal area throughout its ontogeny. A recent genome-wide study showed that among Haplochromine species of Lake Malawi, the utaka group (feeding on zooplankton but exhibiting benthic breeding), which contains *Copadichromis* genus, is closely related to benthic non-Mbuna groups^39^. On the other hand, a previous study has already classified *Cyrtocara moorii* among the non-Mbuna benthic groups^40^, and finally a more recent study, using mitochondrial DNA sequences also grouped *Copadichromis* species and *Cyrtocara moorii* in very closely related clades^41^. It should be noted that taking samples of tissues surrounding the nuchal hump area from the same individuals of *C. moorii* at adult stage, can be an alternative valid approach to filter out age-dependent DE genes as well; particularly, in studies in which closely related species are not easily available.

We found enrichments of multiple biological processes for DE genes in the nuchal hump that involve molecular and cellular responses to stimuli mediated by variety of organic compounds and viral infection. In addition, we found genes involved in regulation of cytokine production to be enriched among these biological processes. It is already known that molecular factors underlying immune and inflammatory responses in soft tissues (e.g. cutaneous tissues) can also lead to local infiltration of inflammatory cells in those tissues and promote proliferation of the surrounding cells (e.g. fibroblasts and dermal cells)^42,43^. An example of such molecular factor can be the components of platelet-derived growth factor (PDGF) signalling, which not only acts as a mitogen in different connective tissues but also participates in viral-derived cell proliferation and fibrotic conditions (excess fibrous connective tissue)^44^. We found a receptor of this signal (*pdgfrb*) to have reduced expression in the nuchal hump, and interestingly, a chromosomal inversion of its homologous gene in humans causes a rare syndrome displaying facial dermal thickening accompanied by local infiltration of inflammatory cells^45^. Moreover, it has been shown that *pdgfrb* plays role in morphogenesis of both soft and hard craniofacial tissues, including tissues forming the forehead area, in mammals and fish^46–49^.

The production of several cytokines had also been implicated in local enhancement of cell proliferation, cellular growth/hypertrophy, excessive extracellular matrix deposition and regulation of lipid accumulation in soft and connective tissues^50–55^. We also found reduced expression of a suppressor of cytokine signalling, *socs2*, in the nuchal hump, which can explain the enrichments of genes involved in cytokine production in the nuchal hump tissues (discussed below). Moreover, the effects of a variety of organic compounds on cell proliferation and extracellular matrix production are already demonstrated in soft and connective tissues such as dermis, and particularly, in relation to inflammatory signals^56–62^. It should be noted that the enrichments for genes responsive to external stimulus might be an indication for a link between formation of nuchal hump and sensing environmental factors during adulthood in *C. moorii*. Indeed, sexually selected exaggerated traits are known to be highly sensitive to the environment^63^. Altogether, these findings suggest involvement of a combination of molecular signals related to cytokines and responses to different organic compounds to be the underlying mechanisms in formation of nuchal hump in young adult of *C. moorii*.

Among the differentially expressed genes, few upstream regulators of transcription, e.g. *ago3*, *dlx5a*, *mycn*, *socs2* and *tcf12*, appeared to have known functions in craniofacial morphogenesis in vertebrates (see Table 3). Three of these regulators, *ago3*, *mycn* and *socs2*, showed reduced expression in the nuchal hump, whereas the two others, *dlx5a* and *tcf12*, had increased expression in this region. The first gene with reduced nuchal hump expression, *ago3*, encodes a member of the Argonaute family of proteins, which play a role in post-transcriptional gene regulation through microRNA-dependent gene silencing. In mammals, it has already been demonstrated that members of the Argonaute family participate in different developmental processes such as skin morphogenesis^64^. A later study in human found genetic association between *ago3* and the emergence of specific craniofacial defects in both hard and soft tissues, among which was the protrusion of forehead^65^. The second gene, *mycn*, encodes a DNA binding protein with a basic helix-loop-helix (bHLH) domain, which regulates apoptosis and is mainly known for its role in the formation of neuroblastoma in mammals^66^. Furthermore, other studies have uncovered the role of *mycn* in craniofacial morphogenesis in mammals, particulalrly, its role in skull and frontal bone ossification^67–69^. However, the effects of *mycn* in development and morphogenesis of craciofacial soft tissues has not been investigated in vertebrates. The third gene, *socs2*, encodes a member of the suppressor of cytokine signaling (SOCS) family, which limits the activity of two growth related signaling pathways, *i.e*. IGF and GH signals^70,71^. *socs2* has been shown to negatively regulate skeletal and dermal growth, and interestingly, its deficiency in mice lead to increased IGF-I production and collagen deposition, which subsequently leads to dermal thickening^72–74^. Furthermore, *socs2* has been shown to downregulate GH signal and affect fat metabolism in adipose tissues in mammals^75^. These observations are consistent with our findings, *i.e*. the reduced *socs2* expression in the nuchal hump could be linked to thickening of the soft tissues through increased activity of abovementioned growth related signals.

The two transcription factors with increased nuchal hump expression, *dlx5a* and *tcf12*, are shown to play role in craniofacial morphogenesis of hard and soft tissues (Table 2). The first gene, *dlx5a*, encodes a member of a homeobox transcription factor gene family which is mainly known for its regulatory role in development of craniofacial skeletal structures in vertebrates^76–79^. In mice, for instance, synergistic regulatory interactions between *dlx5* and other transcription factors have been found to be essential for the formation of the frontal bone^76^. Other studies also showed that *dlx5* is involved in morphogenesis of different facial structures formed by soft tissues in vertebrates^77,80,81^. The potential role of *dlx5a* in development and morphogenesis of non-skeletal craniofacial tissues in fish has not been investigated so far. The second transcription factor, *tcf12*, encodes another member of bHLH E-protein family and plays role in cranio-skeletal development; particularly in the morphogenesis of the frontal bone and cranial vault thickening in mammals^82–84^. Similar to *dlx5a*, the potential role of *tcf12* in development and morphogenesis of non-skeletal craniofacial tissues has yet to be studied in fish.

The delta-like non-canonical Notch ligand 1, *dlk1*, encodes a transmembrane protein, which functions as a regulator of cell growth and is involved in the formation of dermal architecture during skin development^85^. In the same study in mice, it was shown that *dlk1* expression is required for the formation of reticular dermis, hypodermis and adipocyte layer in skin^85^. In human, *dlk1* controls the size of mesenchymal progenitor cells, by inhibiting their differentiation to mature osteoblasts and adipocytes^86^. Genetic changes of *dlk1* in mammals have been reported to cause craniofacial defects such as skeletal growth retardation, protrusion of forehead and thickening of nasal bridge, ears and lips^87,88^. The potential role of *dlk1* in morphogenesis of craniofacial tissues remained elusive in fish, but considering the observations in mammals, its increased nuchal hump expression in *C. moorii* suggests its involvement in thickening of soft tissues in this region by affecting dermal and adipocyte cells.

The other examples of interesting genes with related functions were *cd163*, *dpt* and *pappa* with increased expression, and *btg3* and *fosl1a* with reduced expression in the nuchal hump. *cd163* encodes a member of the scavenger receptor cysteine-rich (SRCR) superfamily which is a histiocytic lineage marker. In human, it has been shown that *cd163* is involved in formation of benign neoplasm of facial soft tissues and xanthogranuloma^89,90^. *dpt* encodes a non-collagenous matrix protein and functions as an enhancer of transforming growth factor-beta (TGF-b) signaling activity, and also plays role in keratinocyte migration, angiogenesis and cell adhesion^91–93^. It is important to note that enhanced activity of TGF-b signaling has been recently shown to induce exaggerated overgrowth of an adaptive flapped nose phenotype in another haplochromine cichlid species^9^. Strikingly, the knockout mice for *dpt* exhibits dramatic reduction in thickness of its dermis, with changes in elasticity of skin, collagen accumulation and subcutaneous adipose tissue^94^. The third gene, *pappa*, encodes an enzyme cleaving IGF-binding proteins, therefore increasing the local bioavailability of IGF-1 in different tissues^95,96^. The knockout mice for *pappa* displays craniofacial defects, including skeletal effects on the frontal bone and skull development^97^. Although the function of these genes have not been investigated in fish, their roles in the above studies in mammals are consistent with their increased expression in the nuchal hump.

The last two genes with reduced expression in the nuchal hump, *btg3* and *fosl1a* (*fra-1*), encode a member of the BTG/Tob family and a leucine zipper protein member of the Fos gene family, respectively. Both genes are involved in regulation of mammalian cell proliferation, as *btg3* acts as an anti-proliferative protein^98^, whereas *fosl1a* can act as inhibitor or enhancer of cell proliferation^99,100^. A chromosomal deletion encompassing *btg3* gene in human has been shown to cause several developmental defects, including effects on the central nervous system and the size of facial elements including forehead, ears and nose^101^. On the other hand, non-functional mutation in *fosl1* in human leads to mainly craniofacial skeletal defects, particularly phenotypes exhibiting fronto-orbital protrusion^102,103^. Moreover, overexpression of *fosl1a* in mice appeared to be suffcient to inhibit the proliferation and differentiation of adipocyte cells, suggesting that its reduced expression in the nuchal hump might be required for the formation of a thicker subcutaneous adipocyte cell layer in this region^104^. Future studies are required to investigate the potential role of *btg3* and *fosl1a* in facial morphogenesis of soft tissues in fish.

## Conclusions

The nuchal hump in cichlids is an enigmatic example of an exaggerated trait whose function remains contentious. It has evolved repeatedly in a several teleosts in males or both sexes. Our findings provide a set of genes known to be involved in adipogenesis, cell proliferation, facial dermal and skeletal development, as well as cytokine, TGF-beta and environmental sensing signals that are driving cichlid nuchal hump formation in *Cyrtocara moorii*. This also highlights the potential developmental plasticity of this phenotype. In the future it would be interesting to investigate if the parallel evolution of nuchal humps in other cichlids and teleosts involves the reuse of the same set of genes.

## Methods

### Fish rearing and tissue sampling

Twelve sibling males of *Cyrtocara moorii* and 7 sibling males of *Copadichromis sp Mbenji*, bred in captivity, were raised in a large tank (approximately 1000 litres) together with similar numbers of females and enough shelters to minimize competition stress. Both species show very similar swimming and feeding behaviour without excessive intra- and inter-species aggression, and they were fed with the same diet adjusted for Malawi cichlids (Tropical Malawi multi-ingredient flakes suitable for omnivorous and herbivorous cichlids). The first sampling group was six males of *C. moorii* at a late juvenile stage (C.m-J), just before protrusion of the frontal/nuchal tissues starting to appear above the eyes (and their sex could be determined) (Fig. 1A). The next sampling was conducted three months later using six young adult males when the nuchal hump was formed with conspicuous protrusion in *C. moorii*, and in parallel, six control samples were taken from comparable nuchal region in young adult males of *C. sp Mbenji*. At the young adult stage both species were showing sexual behaviour such as chasing females, nesting and territorial defending. Prior to dissection, the fish were sacrificed in a solution containing 0.2 g MS-222per 1 L water and the entire soft tissues in the nuchal region (*i.e*. epidermis, dermis and the underlying soft connective tissues) were dissected together for each fish (depicted in Fig. 1A). The entire dissected tissues for each fish were considered as one biological replicate and were immediately submerged into RNAlater (Qiagen) in individual tubes and stored at −20 °C.

### RNA extraction and cDNA synthesis

Total RNA was isolated out of 15 nuchal tissue samples (5 replicates for each group included in the comparison) through the ReliaPrep RNA Tissue Miniprep System Kit (Promega). Each sample contained epidermis, dermis and the underlying soft connective tissues in the specified nuchal region above the eyes (see Fig. 1A). Tissues were immediately submerged into tubes containing 250 µl of lysis buffer mixed with 1-Thioglycerol and 1.4 mm ceramic spheres. The samples were homogenized by FastPrep-24 Instrument (MP Biomedicals, CA, USA) and RNA isolation process was performed according to manufacturer’s ReliaPrep protocol adjusted for fibrous tissues. The isolation method includes a column-based genomic DNA digestion step, followed by several purification steps and excludes any steps of phenol-chloroform phase separation and ethanol-based RNA precipitation. The isolated RNAs were diluted in 30 µl nuclease-free water and quantified with a Nanophotometer (IMPLEN GmbH, Munich, Germany). The RNA qualities were estimated by R6K ScreenTape System on an Agilent 2200 TapeStation (Agilent Technologies) and all samples had above 8 RNA integrity number (RIN). A subset of the isolated RNA from five samples per comparison group was used to perform first strand cDNA synthesis (400 ng of total RNA) following the manufacturer’s protocol of High Capacity cDNA Reverse Transcription kit (Applied Biosystems) and 1:5 times cDNA dilution was used as input for qPCR.

### RNA-Seq library preparation, *de novo* assembly and expression analysis

To recover the list of gene transcripts from the nuchal tissues, we conducted a library preparation step, according to the protocol described by Standard TruSeq Stranded mRNA Sample Prep Kit (Illumina) using an RNA input of 1500 ng per sample. We evaluated the quality of the libraries by D1000 ScreenTape and reagents (Agilent) on a TapeStation 2200 (Agilent). Next, we diluted the libraries to an optimal concentration required for sequencing, which were then pooled equimolar. The RNA-seq was conducted in the NGS Facility at Vienna Biocenter Core Facilities (VBCF, Austria) on one lane of an Illumina HiSeq. 2500, in order to generate 125 bp paired-end reads. The demultiplexing step of the raw reads was performed based on unique barcodes introduced in each sample during library preparation. For each sample, the removal of Illumina adaptors/barcode and initial quality control assessments were applied on the raw reads through the FASTQC tool^105^. The reads with low quality in each sample were filtered out as recommended by standard quality trimming step through the Trimmomatic software^106^. To do this, the filtering criteria was adjusted to retain only the reads with phred +33 quality score of at least 34 for all bases and a minimum length of 50 bp. The *de novo* transcriptome assembly of the nuchal tissues was conducted through the quality trimmed paired-end reads of all samples (using both species) via the Trinity software package^107,108^. This process initially relies on the software Jellyfish^109^ to generate a k-mer catalog, then Trinity package combines 3 independent software modules: Inchworm, Chrysalis, and Butterfly, applied sequentially to process large volumes of RNA-seq reads. First, Inchworm assembles “draft” contigs, then Chrysalis clusters them and bild de Bruijn graphs, and finally Butterfly traces paths through the graphs to reconstruct the final isoform sequences (detailed *de novo* transcriptome assembly using Trinity is described in^107^).

We quantified the transcripts abundances for each sample using the transcriptome assembly and Kallisto, a tool integrated in the Trinity software package, to obtain sample-specific expression level of each transcript^110^. We used transcripts per million transcripts (TPM) measures generated by Kallisto as gene expression unit for the downstream analysis. A weighted trimmed mean of the log expression ratios; trimmed mean of M values (TMM), was used to normalize the data across several samples^111^. The gene expression levels were compared between the nuchal tissues in adult *C. moori* versus juvenile *C. moori*, and adult *C. moori* versus adult *C. sp* Mbenji. For each comparison, the transcripts abundances of all samples involved in the comparison were used to construct a normalized expression matrix using Trinity. Subsequently, differentially expressed transcripts were identified using edgeR package^111–114^ from the R Bioconductor software (R version 3.4.4, R Development Core Team 2018). Significantly differentially expressed genes were extracted through a false-discovery rate (FDR) cutoff of <0.05^115^ and minimum of 2 fold-change to create heatmaps. The dendrograms in the heatmaps were created through hierarchical clustering using the expression values.

In order to annotate the transcripts, we first used TransDecoder software (http://transdecoder.github.io) to identify ORFs with complete coding sequences. TransDecoder identifies candidate protein-coding regions based on nucleotide composition by detecting a minimum length ORF per transcript, computation of a log-likelihood score for each ORF and reporting the longest ORFs per transcript^107^. To maximize sensitivity for capturing ORFs with functional significance, we scanned all ORFs for homology through BLAST tool^116^ against coding sequences (CDS) of Nile tilapia and two other distant teleost fish species *Danio rerio* and *Gasterosteus aculeatus* for further confirmation. Eventually, the gene ontology (GO) term analysis for biological process was conducted with Manteia, an easy to use and recently updated online tool for genomic data processing of a variety of vertebrate species^117^. The GO enrichment criteria was limited to FDR < 0.05 with minimum number of 4 genes per GO term, and while the GO specificity level of 2 was set as cut-off.

### Primer design and qPCR

The qPCR primers for candidate reference and target genes (selected based on the RNA-seq results) were designed after aligning the *de novo* assembled sequences in our study and their homologous sequences from other African cichlid tribes including *Oreochromis niloticus* (Tilapiini), *Neolamprologus brichardi* (Lamprologini), *Callochromis macrops* (Ectodini), and four Haplochromine species; *Maylandia zebra*, *Pundamilia nyererei*, *Ctenochromis horeii* and *Astatotilapia burtoni*^118–120^. This enabled us to identify conserved sequence regions at the exon junctions across East African cichlids using CLC Genomic Workbench, v.9–11 (CLC Bio, Denmark, https://www.qiagenbioinformatics.com/products/clc-genomics-workbench/) and annotated genome of *Astatotilapia burtoni* in the Ensembl database (http://www.ensembl.org). The primers encompass short amplicon size (<200 bp) using Primer Express 3.0 (Applied Biosystems, CA, USA) and their secondary structures and potential dimerization were checked by OligoAnalyzer 3.1 (Integrated DNA Technology) (Supplementary Data 2). We followed the qPCR protocol provided by Maxima SYBR Green/ROX qPCR Master Mix (2X) (Thermo Fisher Scientific, Germany) and the optimal experimental guidelines for qPCR^121^. The qPCR program was started with a 2 min step at 50 °C, a 10 min step at 95 °C, 40 cycles of 15 sec at 95 °C and 1 min at 60 °C (this was adjusted depending on the melting temperature of the primer pairs), and an additional dissociation step at 60–95 °C. The efficiencies of the primer pairs were quantified through the LinRegPCR v11.0 programme^122^ (Supplementary Data 2).

To rank the most stable reference genes, expression stability analyses were performed through three different commonly used software; BestKeeper^123^, NormFinder^124^ and geNorm^125^. For each sample, the average Cq value of the top ranked (most stably expressed) reference genes by the software was considered for normalization of target gene expressions using the following formula: ΔCq _target_ = Cq_target_ − Cq_reference_. In addition, for each gene, the first replicate of the nuchal hump sample from adult C. moorii was specified as calibrator for the calculation of ΔΔCq values (ΔCq_target_ − ΔCq_calibrator_) and relative expression levels (RQ) were calculated by 2^−ΔΔCq^ method^126^. The log-transformed RQ values, ANOVA and Tukey’s HSD post hoc tests were used to calculate the statistically significant differences between the sample groups.

### Ethical approval

Studies of sacrificed fish do not require ethics approval according to the Austrian animal welfare law, as no experiments were carried out with the fish prior to sampling. Fish feeding, breeding and sampling were carried out by Ehsan Pashay Ahi in our certified aquarium facility in Institute of Biology at Karl-Franzens-University Graz according to the Austrian animal welfare law.

## Supplementary information

Supplementary information.

Supplementary information 2.

## Data Availability

All the data represented in this study are provided within the main manuscript or in the Supplementary Materials. In addition, the raw data for RNA-seq are submitted to SRA of NCBI (Accession Number: PRJNA545415).
